# Nonplatinum‐based therapy with Paclitaxel and Capecitabine for advanced squamous cell carcinomas of the anal canal: A population‐based Danish anal cancer group study

**DOI:** 10.1002/cam4.3886

**Published:** 2021-05-07

**Authors:** Christina Glismand Truelsen, Eva Serup‐Hansen, Katrine Smedegaard Storm, Birgitte Mayland Havelund, Camilla Skovhus Kronborg, Karen‐Lise Garm Spindler

**Affiliations:** ^1^ Department of Oncology Aarhus University Hospital Aarhus Denmark; ^2^ Department of Oncology Herlev and Gentofte Hospital Herlev Denmark; ^3^ Department of Oncology at Vejle Hospital University Hospital of Southern Denmark Vejle Denmark; ^4^ The Danish Center for Particle Therapy Aarhus Denmark; ^5^ Department of Experimental Clinical Oncology Aarhus University Hospital Aarhus Denmark

**Keywords:** anal cancer, capecitabine, chemotherapy, metastatic, paclitaxel

## Abstract

**Background:**

First‐line platinum‐based therapy for advanced squamous cell carcinomas of the anal canal (SCCA) implies a risk of substantial side effects, and data on second‐line treatment options are limited. Paclitaxel and Capecitabine are a well‐known regimen with a moderate toxicity profile, but its efficacy has not been evaluated.

**Methods:**

We conducted a retrospective study using Danish Hospital Registers of patients treated with Paclitaxel and Capecitabine for inoperable, recurrent, or advanced metastatic SCCA in Denmark, between January 2000 and July 2018.

**Results:**

A total of 52 patients met the eligibility criteria. Median age was 60.7 years (range 42–83). Efficacy was observed, with an overall response rate in patients receiving first‐line (*N* = 28) and second‐line (*N* = 23) Paclitaxel and Capecitabine of 39.3% (2 with complete responses) and 17.4%, respectively. Median progression‐free survival (PFS) was 4.5 months (95% CI 3.3–5.9) and 3.8 months (95% CI 2.4–5.5) with OS of 6.7 months (95% CI 5.9–8.5) and 5.9 months (95% CI 3.9–14), respectively. Performance status ≥2 and neutrophil to lymphocyte ratio ≥4 were significantly associated with a short PFS.

**Conclusion:**

This study recognizes Paclitaxel and Capecitabine as a potential regimen for advanced SCCA, when recommended first‐line therapy is not feasible or as a potential second‐line treatment after failure of platinum‐based chemotherapy.

## Graphical abstract

Paclitaxel and Capecitabine is recognized for advanced anal cancer when recommended first‐line therapy is not feasible or as second‐line treatment after failure of platinum‐based chemotherapy.

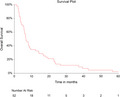

## 1 BACKGROUND

Squamous cell carcinomas of the anus (SCCA) is considered a rare malignancy as it represents 0.5% of all new cancer cases.^1^ Despite the rarity of the condition, there has been an international trend of increased incidences of SCCA resulting in an overall increase in SCCA cases worldwide.^2^, ^3^ SCCAs are commonly confined to the primary site as local (48%) or locoregional (32%) disease, whereas only 13% have metastasized at diagnosis.^1^ Chemoradiotherapy is a well‐established curative treatment for local and locoregional disease with a 5‐year relative survival rate of 82%.^1^ However, some will experience locoregional failure or distant metastasis and the overall prognosis is poor for patients with inoperable, recurrent, or metastatic anal cancer with a 5‐year relative survival rate of approximately 34%.^1^

The combination of a platinum agent (Cisplatin) and a fluoropyrimidine (5‐FU) has until recently constituted the standard first‐line treatment based on phase II trials, smaller retrospective studies, and casuistic rapports with overall response rates varying from 34%–75% with a median progression‐free survival (PFS) survival from 5.8 to 8.0 months.^4^, ^5^, ^6^, ^7^

The first randomized phase II trial, in this setting, the InterACCT, presented at the European Society for Medical Oncology (ESMO) in 2018, compared Cisplatin and 5‐FU with Carboplatin and Paclitaxel as first‐line treatment for advanced SCCA. The results demonstrated a nonsignificant difference in response rate (57.1% vs. 59.0%) and PFS (5.7 vs. 8.1 months). Despite similar response rates, Carboplatin and Paclitaxel demonstrated superior median overall survival (OS) (20 months) and better tolerability, in terms of fewer serious adverse events, compared to Cisplatin and 5‐FU (12.3 months).^8^

Some patients will, however, not be eligible for platinum‐based treatment, and alternative regimens are relevant. Paclitaxel‐based chemotherapy has shown activity in both the chemotherapy‐naive and chemo‐refractory setting in SCCA,^9^, ^10^ and the combination regimen of Paclitaxel and Capecitabine has been investigated as a nonplatinum‐based option for head and neck cancer with relevant efficacy and moderate toxicity reported.^11^ Theoretically, the combination of Taxanes and Capecitabine also offers reasonable predictable efficacy due to the given mechanism of action, and previous studies have found a synergy in vivo, thought to be caused by Taxane up‐regulating dThdPase and thereby enhancing the efficacy of Capecitabine.^12^, ^13^

In Denmark, a combination of Paclitaxel and Capecitabine has consequently been administered for patients with inoperable, recurrent, or metastatic anal cancer either as first‐line therapy for the elderly and/or fragile patients or as second‐line treatment after failure to endure or after progression on a platinum‐based agent. However, outcomes of this regimen have not, to the best of our knowledge, been published in SCCA.

Therefore, a national retrospective analysis of patients treated with Paclitaxel and Capecitabine, in Denmark during the years 2000–2018 was conducted. The aim was to provide clinicians with a background of knowledge to discuss treatment options with patients, in shared decisions making, when first‐line standard recommendation is not feasible or depleted.

## 2 METHODS

In Denmark, the treatment of SCCA is centralized at three National Centers including the University Hospital of Aarhus, University Hospital of Southern Denmark, and Herlev Hospital. Medical files of patients with inoperable, recurrent, or metastatic anal cancer treated with Paclitaxel and Capecitabine were extracted from the National Centers from the period of January 2000 to July 2018. The study was approved by the Danish Patient Safety Authority and the Regional Data Protection Agency.

Inclusion criteria were Histological confirmation of epidermoid anal carcinoma (i.e., squamoucellular, cloacogenic histotype), advanced disease (i.e., inoperable locally recurrent, or metastatic disease), and availability of complete medical records. By using the Danish electronic patient records, data were retrospectively collected and reviewed including patient demographic characteristics, clinicopathological characteristics at initiation of Paclitaxel and Capecitabine, prior or subsequent lines of therapy, and the starting date of treatment and date of progression, as well as whether or not Paclitaxel and Capecitabine was received as first‐line or second‐line therapy. In this analysis, we included all patients commencing the first cycle of therapy.

### 2.1 Chemotherapy

The chemotherapy regimen comprised intravenous Paclitaxel 175 mg/m^2^ at day 1, repeated every third week, and oral administration of Capecitabine 850 mg/m^2^ BID, days 1 to 14. During the treatment period, patients were assessed by thoraco‐abdomino‐pelvic computerized tomography scan with intravenous contrast at every three cycles. Chemotherapy was continued until the progression of cancer, toxicity, or patient wish to discontinue treatment.

### 2.2 Outcomes

Outcome measures were PFS, OS, and objective tumor response rate (ORR). Progression‐free survival was defined as the length of time between the date of initiation of Paclitaxel and Capecitabine to the date of progression. Patients with no event registered were censored at date of death. Overall survival was defined as the length of time between the initiation of Paclitaxel and Capecitabine to date of death from any cause. Additionally, an overall survival was defined from initial date of diagnosis to cause of death from any cause. Patients were censored at date of last follow‐up. The ORR was defined as complete or partial response as per description by treating physicians; retrospective radiological review was not performed for this analysis.

### 2.3 Statistics

The distribution of patient characteristics and baseline variables was presented with descriptive statistics as median and percentages. PFS and OS were statistically analyzed by the Kaplan–Meier method. Prognostic values for selected factors were tested in a univariate model, by log‐rank test. Prognostics factors with cutoff values included, LDH (cutoff; 245 U/L),^14^ neutrophil to lymphocyte ratio (NLR) (cutoff; 4),^15^ albumin (cutoff; 35 g/L),^16^ and hemoglobin (cutoff; 6 mmol/L).

Hazard ratios (HRs) were calculated by Cox proportional hazards regression. Data were calculated with 95% confidence intervals (CI) and a *p*‐value <0.05 was considered statistically significant. NCSS software version v20.0.1 was used to perform statistical analyses.

## 3 RESULTS

A total of 52 patients met the eligibility criteria. Demographic and clinical characteristics are reported in Table 1. The median age at primary diagnosis of SCCA was 60.7 years (range 42–83), with a predominance of women (67.3%). In this cohort, only 10 patients (19.2%) were classified as being in performance status (PS) 0, the majority in PS 1 (26 patients (50%) and PS 2 (12 patients (23.1%)). A total of 10 (19.2%) had inoperable, locally advanced disease, whereas the remaining 42 (80.8%) presented with distant metastases. Lymph node or liver metastases were the most frequent metastatic sites, 65.4%, and 42.3% respectively.

**TABLE 1:** Patients pretreatment characteristics

Characteristics	Value *n* = 52 (%)
Gender	
Female	35 (67.3)
Male	17 (32.7)
Median age (range), year	60.7 (42–83)
Immunodepression	
HIV positive	2 (3.8)
HIV no known history	50 (96.2)
HPV (human papillomavirus)	
Positive	20 (38.5)
Negative	3 (5.8)
Unknown	29 (55.8)
Smoking	
Never smoker	11 (21.2)
Former smoker	17 (32.7)
Current smoker	10 (19.2)
Unknown	14 (26.9)
ECOG, performance status	
PS: 0	10 (19.2)
PS: 1	26 (50.0)
PS: 2	12 (23.1)
PS: 3	0 (0)
Unknown	4 (7.7)
Extent of disease	
Locally advanced	10 (19.2)
Metastatic	42 (80.8)
Sites of distant metastasis	
Liver	22 (42.3)
Lymph nodes	34 (65.4)
Lungs	20 (38.5)
Other	8 (15.4)

In Table 2 prior therapies are reported. The majority, 47 (90.4%) had received prior radiotherapy, of those 23 received concomitant chemotherapy, with the most frequent concomitant radiosensitizing regimen being Cisplatin and 5‐FU (69.6%). In fewer cases, Cisplatin was administered as monotherapy, (26.1%). A total of 11 patients had previously received neoadjuvant chemotherapy in an attempt of a curative strategy (21.2%).

**TABLE 2:** Treatment characteristics

Variable	Patients, *n* (%)
Line of Paclitaxel and Capecitabine	
First‐line	28 (53.8)
Second‐line	23 (44.2)
Third‐line	1 (1.9)
Prior radiotherapy	
Yes	47 (90.4)
No	4 (7.7)
Unknown	1 (1.9)
Prior neoadjuvant chemotherapy	
Yes	11 (21.2)
No	41 (78.8)
Prior concomitant chemoradiotherapy	
Yes	23 (44.2)
Cisplatin monotherapy	6 (26.1)
Cisplatin and 5‐fluoruracil	16 (69.6)
5‐FU or capecitabine	1 (4.3)
No	29 (55.8)
Prior palliative chemotherapy	
Yes	24 (46.2)
CILF (Cisplatin, Ifosfamid, 5‐FU)*	24 (100)
Carboplatin/Paclitaxel	1 (4.2)
No	28 (53.8)

* The CILF regimen was used as intensified chemotherapy for advanced disease. Data will be presented separately.

The combination of Paclitaxel and Capecitabine was prescribed as first‐line treatment in 53.8% of patients (*n* = 28), as second‐line in 44.2% (*n* = 23), and third line in 1.9% (*n* = 1). All patients, receiving second‐line treatment with Paclitaxel and Capecitabine, had previously been treated with a Cisplatin‐based regimen. Patients received a median of 6 and 4.4 cycles of treatment during first‐line and second‐line therapy, respectively.

Objective responses were observed in 15 patients (28.8%). Of those, 2 (3.8) achieved a complete response and 13 (25%) a partial response, whereas an additional 25% obtained stable disease. The antitumor activity is reported in Table 3. Comparing first‐ and second‐line treatment, responses were observed in 39.3% and 17.4%, respectively.

**TABLE 3:** Antitumor Activity in the overall population, as first‐line treatment and as second‐line treatment

Variable	Overall population, *n* = 52 (%)	1‐line, *n* = 28 (%)	2‐line, *n* = 23 (%)
Objective response rate	15 (28.8)	11 (39.3)	4 (17.4)
Overall response			
Complete response	2 (3.8)	2 (7.1)	0 (0)
Partial response	13 (25.0)	9 (32.1)	4 (17.4)
Stable disease	13 (25.0)	6 (21.4)	6 (26.1)
Progressive disease	16 (30.8)	7 (25.0)	9 (39.1)
Nonevaluable	8 (15.3)	4 (14.3)	4 (17.4)

**FIGURE 1:**
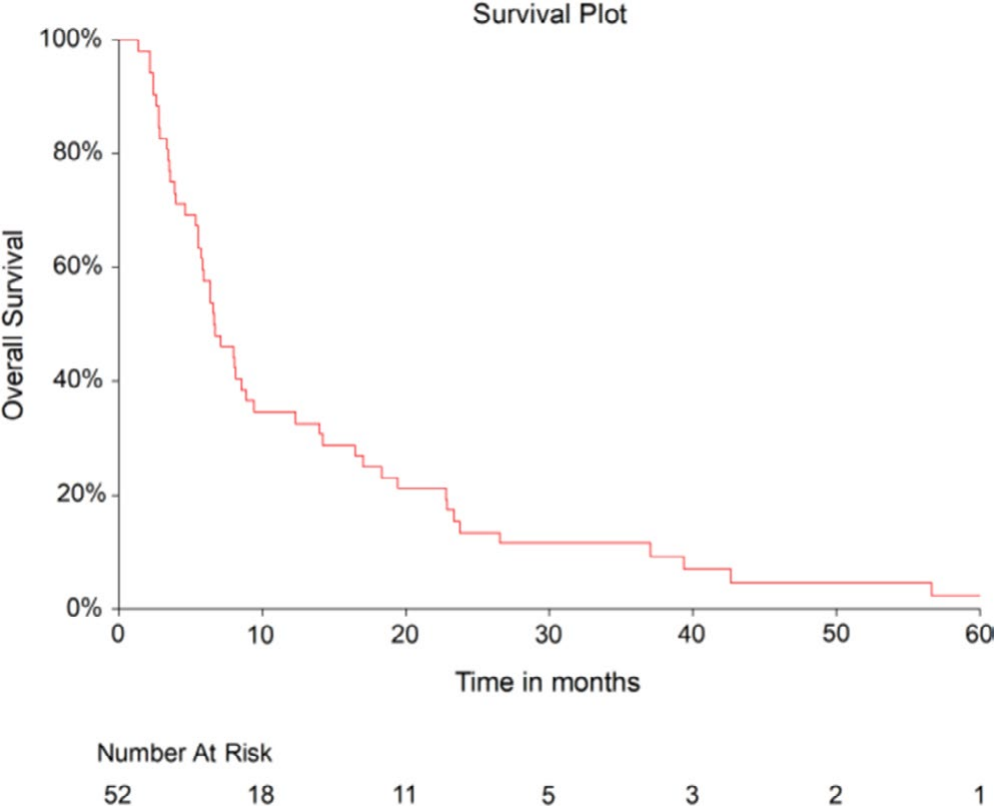
Kaplan–Meier plot of overall survival in the 52 patients treated with paclitaxel and capecitabine

Median PFS was 4.4 months (95% CI, 3.0–5.5) in the overall population and 4.5 months (95% CI 3.3–5.9) and 3.8 months (95% CI 2.4–5.5) in patients who received first‐line and second‐line treatment, respectively. Median OS was 6.7 months (95% CI, 5.7–8.5) in the overall studied population (Figure 1) and 6.7 months (95% CI 5.9–8.5) for patients receiving first‐line treatment. For second‐line treatment, OS was 5.9 months (95% CI 3.9–14). The median OS estimated from the date of initial diagnosis was 31.2 months (95% CI, 27.5–36.4) corresponding to 2.6 years.

Univariate analysis of potential pretreatment clinical and biochemical prognostic parameters is presented in Table 4. Performance status ≥2 was significantly associated with a worse PFS (HR, 2.87; 95% CI, 1.15–7.19; *p* = 0.009). Patients in PS = 0 had a PFS of 7.5 months (95% CI, 4.4–10.9) and an OS of 22.8 months (95% CI, 8.9–26.5) compared to a PFS of only 3.3 months and OS of 5.9 months for patients in PS = 2. Patients with low levels of hemoglobin had a shorter OS (HR, 0.47; 95% CI, 0.21–1.04, *p* = 0.01). Further, NLR greater than the cutoff of 4 was significantly associated with a worse PFS (HR, 2.03; 95% CI, 1.90–3.78; *p* = 0.02) and a worse OS (HR, 2.22; 95% CI, (1.18–4.17; *p* = 0.01).

**TABLE 4:** Univariate analysis of prognostic factors for progression‐free survival and overall survival

Variable	PFS HR (95% CI)	PFS *p* value	OS HR (95% CI)	OS *p* value
Gender				
Female	—	—	—	—
Male	1.00 (0.56–1.79)	0.99	0.8 (0.38–1.20)	0.20
Age				
< 65 years	—	—	—	—
> 65 years	1.31 (0.73–2.35)	0.35	1.07 (0.60–1.90)	0.83
Performance status				
0	—	—	—	—
1	1.40 (0.70–2.80)	0.36	1.93 (0.98–3.83)	0.08
2	2.87 (1.15–7.19)	0.01*	2.06 (0.86–4.93)	0.09
Smoking				
Never	—	—	—	—
Former	1.65 (0.68–4.04)	0.24	0.88 (0.37–2.07)	0.77
Current	1.41 (0.67–2.96)	0.37	1.44 (0.68–3.05)	0.35
Liver metastases				
No	—	—	—	—
Yes	1.67 (0.92–3.02)	0.06	0.56 (0.22–1.43)	0.29
Extension				
Locoregional	—	—	—	—
Distant	0.85 (0.41–1.77)	0.65	0.76 (0.36–1.62)	0.43
Number of sites				
0	—	—	—	—
1	0.42 (0.10–1.88)	0.10	0.52 (0.13–2.05)	0.22
2	0.90 (0.29–2.79)	0.85	0.99 (0.33–2.97)	0.98
3	0.69 (0.19–2.53)	0.54	0.61 (0.16–2.33)	0.42
4	2.53 (0.12–54.06)	0.39	6.00 (0.07–525.71)	0.06
LDH				
< 245	—	—	—	—
> 245	1.20 (0.61–2.36)	0.57	1.58 (0.74–3.34)	0.17
Albumin				
< 35	—	—	—	—
> 35	0.99 (0.50–1.97)	0.98	0.75 (0.36–1.56)	0.41
Hemoglobin				
< 6	—	—	—	—
> 6	0.67 (0.33–1.36)	0.21	0.47 (0.21–1.04)	0.01*
NLR				
< 4	—	—	—	—
> 4	2.03 (1.90–3.78)	0.02*	2.22 (1.18–4.17)	0.01*

Abbreviations: CI, confidence interval; HR, hazard ratio; NLR, neutrophil to lymphocyte ratio; OS, overall survival; PFS, progression‐free survival.

* *p* < 0.05.

## 4 DISCUSSION

The optimal management of inoperable, locally advanced, or metastatic SCCA has undergone debate, due to insufficient high‐quality evidence. Until now, clinicians have based their treatment decisions on suboptimal evidence, consisting of smaller retrospective studies, casuistic rapports, and smaller phase I and II studies, often presenting heterogeneous treatment regimens. Attributable to this is the low incidence of advanced SCCA cases, due to the high success rates of definitive chemoradiotherapy. Especially second‐line options are rarely presented in the literature.

In this population‐based study, we report the outcome after treatment with Paclitaxel and Capecitabine for advanced SCCA in Denmark during 2000–2018. The regimen is well known and considered feasible with only moderate toxicity in different settings,^11^ but reports on efficacy in SCCA are lacking. The aim was to generate a background of knowledge for clinicians to discuss alternative treatment strategies, in shared‐decision making, when the recommended guideline of first‐line treatment is not feasible or depleted. We report unselected real‐world experience with this regimen in patients with comorbidity or fragility or after failure of first‐line treatment. This is, to the best of our knowledge, the first study to report outcomes of this combination regime as first‐ and second‐line treatment for advanced SCCA.

Previous retrospective studies have reported on the use of paclitaxel‐based regimes, primarily in combination with carboplatin or as a single agent. Objective response rates vary between 33% and 69% as first‐line treatment, and when treatment was administered as second‐line, the regime appeared less favorable.^6^, ^7^, ^17^, ^18^, ^19^

In our cohort, the median age at initiation of Paclitaxel and Capecitabine was relatively young, with a minor fraction of patients classified as PS 0 and a relatively high frequency of patients in poor PS of 2. As anticipated, this is in contrast with the cohort of patients treated according to the InterACCT trials with 93% in PS 0–1 and only 7% in PS 2. Of note, the patients in this cohort in PS 0 (19%) presented with a mOS of 22.8 months compared to a mOS of 5.9 months in PS = 2 (23%), and PS = 2 was significantly associated with a worse PFS. A previous study indicated that survival was approximately halved for each worsening performance level^20^ underlining the importance of patients general clinical status for treatment decisions.

In our report, a total of 28 patients received Paclitaxel and Capecitabine as first‐line therapy. A platinum agent and 5‐FU had been evaluated for all patients treated in the first‐line setting but was deemed not feasible due to contraindications, comorbidity, poor performance status, or patient wish for a lesser toxic treatment. The ORR was 39.3% (two complete responders) with an additional 21% obtaining stable disease. Progression‐free survival was 4.5 (95% CI 3.3–5.9) months, and OS was 6.7 months (95% CI, 5.7–8.5). In comparison, retrospective first‐line studies of Cisplatin and 5‐FU presented ORR of 34%–75%, PFS of 5.8–8.0 months, and OS of 12–34.5 months.^4^, ^5^, ^6^, ^7^ Thus, relevant activity was observed, and our data indicate that the regimen in this study can be considered for patients with conditions where lesser toxicity is demanded and platinum‐based treatments are not feasible.

This study is the largest retrospective study, estimating the efficacy of Paclitaxel combined with Capecitabine as second‐line treatment. An ORR was observed in 17.4% and a further 26% obtained disease stabilization. Progression‐free survival was 3.8 months (95% CI 2.4–5.5), and OS was 5.9 (95% CI 3.9–14) months. These observations are comparable to the results from studies investigating other cytotoxic agents as second‐line treatments. A relatively recent study assessed the benefits of Mitomycin and 5‐FU after failure of Cisplatin and 5‐FU in 19 patients. Similar ranges were seen with an ORR of 26%, median PFS of 3 months, and OS of 7 months.^21^

The univariate analyses of prognostic factors pointed out NLR ≥4 as a predictor of worse PFS and OS. This is substantiated by a pan‐cancer study comprising 40.559 cancer patients where NLR was found to be associated with adverse OS.^15^ Regards to SCCA a study assessed whether or not NLR could be used as a predictor of locoregional recurrence and found NLR significantly higher in lymph node positive disease and in patients developing recurrence.^22^ Prognostic factors in small sample sizes must be interpreted with caution. However, PS and NLR have shown a prominent role.

This study is limited by its retrospective nature but constitutes a population‐based, national cohort describing real‐world data. This also precludes collection of valid toxicity data, but as described above, the regimen is known to be feasible with moderate toxicity in other settings. In Denmark Cisplatin is often used instead of Mitomycin concomitantly with radiotherapy and in first‐line treatment for advanced disease. This increases the need for nonplatinum options, which might not be applicable for centers using Mitomycin as the standard of care.

For the rare group of advanced SCCA, this is one of the larger studies concerning first‐ and second‐line therapy for heavily pretreated and fragile patients and therefore a valuable source of information for clinicians in the tailoring of treatment for patients not eligible for first‐line platinum‐based therapy. A randomized study, comparing Paclitaxel and Capecitabine with the current standard first‐line, should however be undertaken to demonstrate the efficacy and report on toxicity.

## 5 CONCLUSION

In conclusion, the management of SCCA has been illuminated in recent years with intriguing studies in pipeline, but not all patients are eligible for intensive cytotoxic treatment or experimental options. Paclitaxel and Capecitabine are a reasonable option of therapy for patients not eligible for or after the failure of first‐line treatment with PS and NLR as predictive factors of worse outcomes.

## CONFLICT OF INTEREST

The authors declare no competing interests.

## ETHICS APPROVAL AND CONSENT TO PARTICIPATE

The study was approved by the Danish Patient Safety Authority and the Regional Data Protection Agency. Patients representatives have not been involved in the present study design, due to its retrospective nature.

## CONSENT FOR PUBLICATION

All authors have read the paper and given consent for publication.

## Data Availability

The data analyzed in the current study are available upon reasonable request from the corresponding author.
